# Persisting gastrointestinal symptoms and post-infectious irritable bowel syndrome following SARS-CoV-2 infection: results from the Arizona CoVHORT

**DOI:** 10.1017/S0950268822001200

**Published:** 2022-07-08

**Authors:** Erika Austhof, Melanie L. Bell, Mark S. Riddle, Collin Catalfamo, Caitlyn McFadden, Kerry Cooper, Elaine Scallan Walter, Elizabeth Jacobs, Kristen Pogreba-Brown

**Affiliations:** 1 Department of Epidemiology & Biostatistics, Mel and Enid Zuckerman College of Public Health, University of Arizona, Tucson, Arizona, USA; 2 Department of Internal Medicine, Reno School of Medicine, University of Nevada, Reno, Nevada, USA; 3 School of Animal and Comparative Biomedical Sciences, University of Arizona, Tucson, Arizona, USA; 4 Department of Epidemiology, Colorado School of Public Health, University of Colorado Anschutz Medical Campus, Aurora, Colorado, USA; 5 University of Arizona Cancer Center, Tucson, Arizona, USA

**Keywords:** COVID-19, post-infectious irritable bowel syndrome, gastrointestinal, post-acute sequelae of COVID-19

## Abstract

In this study, we aimed to examine the association between gastrointestinal (GI) symptom presence during severe acute respiratory syndrome coronavirus 2 (SARS-CoV-2) infection and the prevalence of GI symptoms and the development of post-infectious irritable bowel syndrome (PI-IBS). We used data from a prospective cohort and logistic regression to examine the association between GI symptom status during confirmed SARS-CoV-2 infection and prevalence of persistent GI symptoms at ≥45 days. We also report the incidence of PI-IBS following SARS-CoV-2 infection. Of the 1475 participants in this study, 33.8% (*n* = 499) had GI symptoms during acute infection. Cases with acute GI symptoms had an odds of persisting GI symptoms 4 times higher than cases without acute GI symptoms (odds ratio (OR) 4.29, 95% confidence interval (CI) 2.45–7.53); symptoms lasted on average 8 months following infection. Of those with persisting GI symptoms, 67% sought care for their symptoms and incident PI-IBS occurred in 3.0% (*n* = 15) of participants. Those with acute GI symptoms after SARS-CoV-2 infection are likely to have similar persistent symptoms 45 days and greater. These data indicate that attention to a potential increase in related healthcare needs is warranted.

## Introduction

Irritable bowel syndrome (IBS) is a chronic intestinal disorder that causes abdominal pain, diarrhoea and/or constipation resulting in a significant impact to a person's quality of life. Prevalence within the U.S. has been estimated to be 10–15% within adults [1]. Post-infectious IBS (PI-IBS), has been linked to acute bacterial, protozoal and viral infections [2]. However, little is known about PI-IBS following acute coronavirus disease 2019 (COVID-19) infection. Infection with severe acute respiratory syndrome coronavirus 2 (SARS-CoV-2) includes a wide range of symptoms including fever, cough, shortness of breath, fatigue and in some cases gastrointestinal (GI) symptoms. Estimates of GI symptoms during acute infection vary – one meta-analysis showed that GI symptoms are present in <10% of COVID-19 patients [3]; while other multicentre cohort studies have found GI symptoms during acute infection present in as many as 61% of participants [4]. While not completely understood, the persistence of GI symptoms after acute infection can trigger an intestinal inflammatory process [5] and/or gut dysbiosis [6] which are hallmark characteristics for some functional GI disorders. Given the scale of the ongoing pandemic and the notable association of PI-IBS after viral GI infections [7–9], it is important to understand if there is an epidemiologic association which might inform an important chronic consequence of COVID-19 associated with long-term GI disorders [10].

Initial results (May 2020–October 2021) from a prospective study in Arizona (CoVHORT) indicated GI symptoms were present in 18.3% (≥30 days), 17.2% (30–59 days) and 19.0% (≥60 days) of participants experiencing post-acute sequelae of COVID-19 (PASC) [11]; the prevalence of PASC at 30 days post-infection was 68.7% (95% CI 63.4–73.9). In a single study from Italy COVID-19 positive patients with diarrhoea during acute infection were compared to COVID-19 negative patients for GI symptoms at 5 months after acute COVID-19 infection and found that GI complaints and somatisation were greater, although mild, in case patients [12]. Research on persisting GI symptoms and development of functional GI disorders after COVID-19 infection is new, thus the objectives of this study were to: (1) examine the association between GI symptom presence during acute infection and the prevalence of GI symptoms ≥45 days post-acute infection in positive COVID-19 adult participants of the Arizona CoVHORT study and (2) describe the symptom presentation and healthcare utilisation of participants who report GI symptoms ≥45 days post-acute infection and for participants who develop post-infectious IBS.

We hypothesise that persons with GI symptoms during acute infection will have a higher prevalence of GI symptoms post-acute infection and have PI-IBS more often, compared to persons who did not have GI symptoms during acute infection. In addition to previously reported evidence showing that GI symptoms can persist following SARS-CoV-2 [11, 12], other sequelae of COVID-19 include neurological symptoms, fatigue and brain fog [13]. These symptoms might also influence the gut-brain axis – a pathway present in disorders of gut-brain interaction like IBS [14]. If persistent GI symptoms or PI-IBS are significant post-acute sequelae of COVID-19, the implications for decreased personal quality of life and increased financial burden from additional PI-IBS case-patients could be considerable [15].

## Methods

### Study design and setting

Details regarding the study design of CoVHORT have been published previously [16]. Arizona residents can enrol in CoVHORT after completing a routine public health surveillance interview following SARS-CoV-2 infection, after free diagnostic testing at sites throughout Arizona, through collaborations with other COVID-19 research studies, or through targeted recruitment campaigns (e.g. phased mailing, social media, listservs). Both infected and non-infected individuals can enrol, at any time. Participants must be able to complete online surveys in English or Spanish, and be a resident of Arizona. Participant recruitment began in May 2020, immediately following approval by the University of Arizona Institutional Review Board (Protocol #2003521636). Recruitment in CoVHORT is ongoing to track changes in health conditions, and to identify new case-patients and re-infections over the course of the pandemic in Arizona.

In this study, we included adults (≥18 years) within the Arizona CoVHORT who had a positive COVID-19 infection confirmed through a self-reported positive polymerase chain reaction (PCR) test for SARS-CoV-2. We defined two groups of participants, one group who had GI symptoms during acute infection, and a second group that did not have GI symptoms during acute infection. We consider ‘acute infection’ to represent the initial illness period following SARS-CoV-2 infection where COVID-19 signs or symptoms are present, indicating viral replication and an initial immune response [17, 18]; COVID-19 symptoms may appear 2–14 days after exposure to the virus. GI symptoms were defined as diarrhoea (3 or more loose or looser than normal stools in a 24-h period), nausea or vomiting self-reported during their acute infection. All data were acquired through self-reported, online surveys completed between May 2020 and October 2021.

### Participants and data collection

Participation in the Arizona CoVHORT included a series of online questionnaires every 6 weeks or 3 months depending on current symptom or COVID-19 status, and topic-specific questionnaires based on eligibility criteria. This timeline ensures participants are regularly engaged in the study while still providing enough time between surveys to allow for changes in health status to occur. After informed consent, participants completed a baseline questionnaire including demographics, COVID-19 testing status and symptoms, potential exposures and general health and wellbeing questions. Participants who reported a positive SARS-CoV-2 infection lab result were asked about their symptoms during the acute infection. Those people who reported GI symptoms during their acute infection were asked to complete the Rome IV diagnostic questionnaire for IBS at 6-weeks, 3- and 6-month timepoints following their first survey completion [19]. We excluded participants that reported pre-existing GI conditions including: IBS, inflammatory bowel disease including Crohn's disease or ulcerative colitis, chronic diarrhoea, chronic constipation, colitis, dyspepsia or reflux.

### Measures

**Fig. 1.**
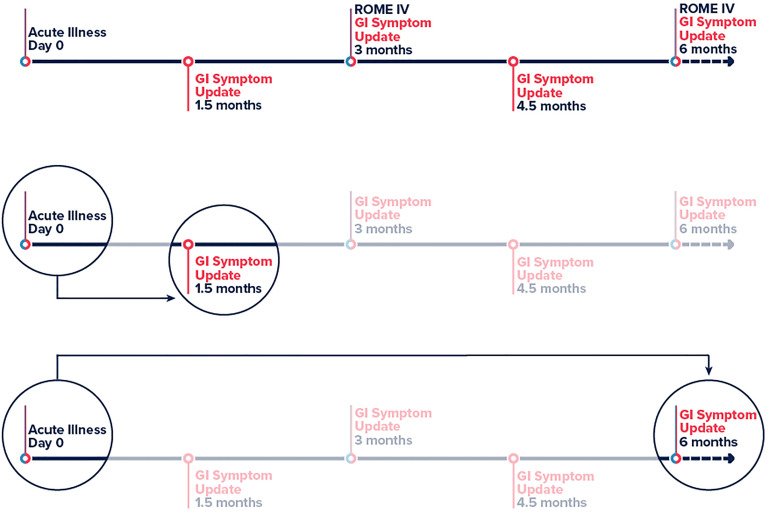
Timeline of participation in the Arizona CoVHORT. Legend: Abbreviations: GI, gastrointestinal. Line 1 is the full participant timeline in CoVHORT with survey data timepoints highlighted and used in the analysis. Line 2 shows the primary analysis of day 0 to persisting GI symptoms at ≥45 days. Line 3 shows the timeline for the second sensitivity analysis in which we change the outcome definition from 45 days (line 2) to ≥180 days.

The primary exposure of interest was GI symptoms defined as diarrhoea (3 or more loose or looser than normal stools in a 24-h period), nausea or vomiting during acute infection of COVID-19. The primary outcome was persistent GI symptoms ≥45 days post post-acute COVID-19 infection. We used the time from positive test result (or onset date when test date was unavailable) to report of new or continuing GI symptoms to determine the 45 day minimum cutoff for persisting GI symptoms. The timeline for a participant in the Arizona CoVHORT is shown in Figure 1. For our secondary analysis, PI-IBS via the Rome IV is defined as recurrent abdominal pain on average at least 1 day/week in the last 3 months, with symptom onset at least 6 months before, associated with two or more of: (1) being related to defecation, (2) associated with a change in stool frequency and (3) associated with a change in stool appearance or form [20, 21].

Covariates included age, gender (female, male, non-binary, transgender female, transgender male), ethnicity (Hispanic, non-Hispanic), acute infection severity (1–10 point self-assessment scale) and stress and emotional wellness using the Perceived Stress Scale (PSS-10) [22]. Categories of gender were recoded to sex assigned at birth (female, male) to address issues with small cell sizes. Acute infection severity used the following question, ‘How would you characterise the severity of your illness? 1 – very mild, easy to continue daily activities to 10 – extremely serious’, with higher scores indicating a more severe illness. Due to the link between stress and the development of IBS [23], we included the PSS, a 10-item stress assessment instrument with a range of 0–40. Higher PSS scores indicate higher stress levels and lower emotional wellness (score 0–13 indicates low stress, 14–26 indicates moderate stress, 27–40 indicates high stress).

### Statistical methods

Descriptive statistics were calculated and stratified by GI symptom status during acute infection. We used *χ*^2^ and *t* tests, or nonparametric equivalents (i.e. Fisher's exact test) as appropriate, to assess differences between case groups, and among those who completed the Rome IV survey or not. We used logistic regression to examine the association between GI symptom status during acute infection and prevalence of persistent GI symptoms at ≥45 days, reporting unadjusted and adjusted odds ratios (OR) and 95% confidence intervals (CI). Due to the low number of participants with our outcome (*n* = 45), we restricted the covariates in the model to include those with the lowest level of missingness. Ultimately, we included sex, age and perceived stress via the PSS score in the final adjusted logistic regression model. We assessed collinearity using Pearson correlation coefficients, and linearity in the log-odds using LOESS plots for continuous variables (age, and PSS); both appeared linear and were kept continuous. We described the frequency and 95% CIs of GI symptom presentation at ≥45 days, as well as the health experience of participants who developed PI- IBS. We performed two sensitivity analyses. First, to account for missingness in the perceived stress variable (25% missing), we imputed the sample average item score for each item that was missing, as long at least 5 items in the score were answered by the participant, which is a common approach for missing items [24]. If PSS was still missing, we used single imputation of the 6-week survey PSS score for participants in the initial CoVHORT enrolment period in which PSS was not asked during their first survey, but rather during the 6-week survey. Second, we redefined the primary analysis outcome to GI symptom presentation ≥180 days and ran the primary analysis, adjusted for the same covariates. We performed all statistical analyses using Stata 17 (StataCorp LP, College Station, TX).

## Results

**Fig. 2.**
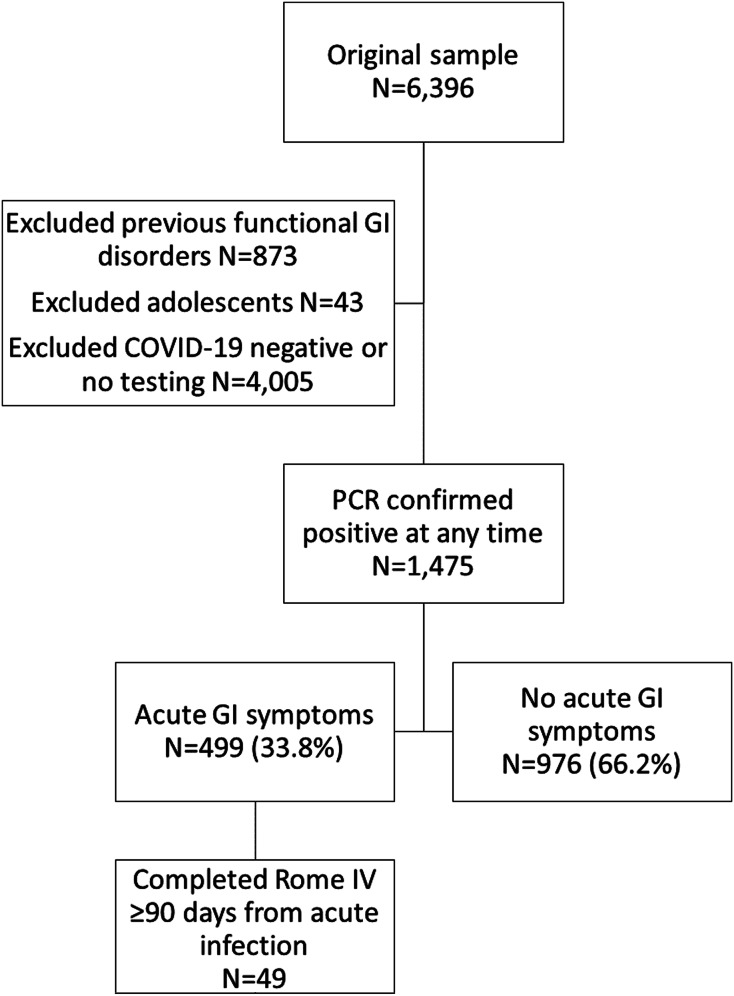
Participant flow diagram, May 2020–October 2021. Legend: Abbreviations: GI, gastrointestinal. Data is from the Arizona CoVHORT study. COVID-19 case status was determined based on a confirmatory polymerase chain reaction positive test.

The Arizona CoVHORT enrolled 6396 participants in the study from May 2020–October 2021. After excluding participants with a previous functional GI disorder, adolescents, and those with no or negative COVID-19 testing, our sample included 1475 participants (Fig. 2). Most of these individuals did not have GI symptoms during their acute infection (66.2%). In general, those with GI symptoms during their acute infection were slightly younger (42.7 *vs.* 45.0 years, *P* = 0.01) and more likely to be female (73.5% *vs.* 62.6%, *P* < 0.01) than those without GI symptoms (Table 1). Those with GI symptoms also indicated higher perceived stress via the PSS (18.7 *vs.* 16.5, *P* < 0.01) and a more severe acute infection (5.9 *vs.* 4.3, *P* < 0.01).

**Table 1.** Characteristics of adult Arizona CoVHORT participants who tested positive for COVID-19, May 2020–October 2021 stratified by acute gastrointestinal symptom status (*n* = 1475)

Characteristic	No GI symptoms (*n* = 976), *n* (%)	GI symptoms (*n* = 499), *n* (%)	*P*-value^a^
Age (years), mean (s.d.)	45.0 (15.9)	42.7 (15.6)	**0**.**01**
Gender			**<0**.**01**
Female	611 (62.6)	367 (73.5)	
Male	358 (36.7)	129 (25.9)	
Non-Binary	5 (0.5)	2 (0.4)	
Transgender female	1 (0.1)	1 (0.2)	
Transgender male	1 (0.1)	0 (0)	
Ethnicity			0.07
Hispanic	194 (20.3)	120 (24.5)	
Non-Hispanic	760 (79.7)	369 (75.5)	
Highest education level			**0**.**02**
9–12th grade, or GED	29 (3.7)	18 (5.3)	
Some college (1–3 years)	179 (23.0)	105 (30.7)	
College (4 years or more)	244 (31.4)	105 (30.7)	
Post-graduate education	326 (41.9)	114 (33.3)	
Annual household income			0.62
$0–$74 999	281 (41.2)	135 (42.9)	
≥$75 000	401 (58.8)	180 (57.1)	
Perceived Stress Scale^b^, mean (s.d.)	16.5 (7.9)	18.7 (8.2)	**<0**.**01**
Acute Infection Severity^c^, mean (s.d.)	4.3 (2.4)	5.9 (2.3)	**<0**.**01**

Abbreviations: GI, gastrointestinal; s.d., standard deviation; GED, General Education Degree.

a Significant differences between the groups were assessed using *χ*^2^, *t* tests, or nonparametric alternatives (Fisher's exact for gender) as appropriate, *P* < 0.05 is considered significant and is bolded.

b The Perceived Stress Scale ranges from 0–40, scores 0–13 indicate low stress, 14–26 indicate moderate stress, and 27–40 indicate high stress.

c Acute infection severity ranges from 0–10, with 1 indicating very mild illness, and 10 indicating extremely serious illness.

Participants reported persistent GI symptoms on average 8 months (Mean: 226.7 days, Median: 221, s.d.: 99.5, Range: 47–422 days) from their acute infection timepoint. Of the 499 participants with acute GI symptoms, 45 (9.0%) had persisting GI symptoms, 450 (90.2%) reported no persisting GI symptoms, and 4 (0.8%) were lost to follow-up. Of the 976 participants without acute GI symptoms, 23 (2.4%) had persisting GI symptoms, 931 (95.4%) reported no persisting GI symptoms, and 22 (2.3%) were lost to follow-up. Of the 68 participants who reported GI symptoms ≥45 days post-acute COVID-19 infection, they reported various symptoms including diarrhoea (*n* = 17, 25.8%), constipation (*n* = 9, 13.6%), and acid reflux or heartburn (*n* = 8, 12.1%). Less reported symptoms included feeling of not emptying bowels (*n* = 3, 4.5%), feeling full quickly while eating (*n* = 3, 4.5%), anorexia or weight loss >10 pounds (*n* = 2, 3.0%), and anxiety or depression (*n* = 5, 7.6%). Forty-two participants (61.7%) sought care from a provider for these symptoms.

In Table 2 we show the results of the logistic regression model conducted to examine the association between GI symptom status during acute infection and the prevalence of GI symptoms ≥45 days post-acute infection. Cases with acute GI symptoms had an odds of persisting GI symptoms 4 times higher than cases without acute GI symptoms (OR 4.04, 95% CI 2.42–6.77). After adjusting for age, sex and perceived stress during acute infection, the results remain similar (OR 4.29, 95% CI 2.45–7.53). Sensitivity results were similar. After imputing missing perceived stress scores, cases with acute GI symptoms had an odds of persisting GI symptoms 3.63 (95% CI 2.09–6.28) times higher than cases without acute GI symptoms. After redefining the outcome to GI symptom presentation to ≥180 days post-acute infection, the results remain similar to the primary adjusted analysis albeit with a larger CI due to participant dropout (OR 4.27, 95% CI 1.62–11.21).

**Table 2.** Logistic regression odds ratios and 95% confidence intervals for the relationship between gastrointestinal symptoms during acute infection and gastrointestinal symptoms ≥45 days post-acute infection in adult Arizona CoVHORT participants who tested positive for COVID-19 (May 2020–October 2021)

Acute infection symptoms	≥45 days GI symptoms n (%)*	Unadjusted OR (95% CI)	Adjusted^a^ OR (95% CI)	Sensitivity analysis adjusting for missingness^b^ OR (95% CI)	Sensitivity analysis with new GI definition^c^ OR (95% CI)
GI symptoms (*n* = 499)	45 (9.0)	4.04 (2.42–6.77)	4.29 (2.45–7.53)	3.63 (2.09–6.28)	4.27 (1.62–11.21)
No GI symptoms (*n* = 976)	23 (2.3)	–	–	–	–

Abbreviations: GI, gastrointestinal; OR, odds ratio; CI, confidence interval.

*Proportions take into account lost to follow-up: *n* = 4 for GI symptoms, *n* = 22 for no GI symptoms.

a Adjusted for age, sex and perceived stress via the Perceived Stress Scale (PSS).

b Adjusted for the same covariates. Missing perceived stress values were imputed with the subject mean for each item if less than 5 items were missing, or with the 6-week PSS if enrolment was in the original CoVHORT survey when PSS scores were not asked at baseline.

c Adjusted for the same covariates. New outcome definition of GI symptom presentation to ≥180 days post-acute infection.

In a post-hoc analysis, suggested by a reviewer of the manuscript, we found that the odds of persisting GI symptoms was modified by the presence of other, non GI, pre-existing chronic conditions (*p*_interaction_ = 0.001). For individuals without pre-existing chronic conditions the odds of persisting GI symptoms was 8.34 (95% CI 2.91–23.94) times higher than cases without acute GI symptoms after adjusting for age, sex and perceived stress (Table 3). For cases with at least one pre-existing condition (other than pre-existing GI conditions), the odds of persisting GI symptoms was 3.18 (95% CI 1.62–6.26) times higher after adjusting for age, sex and perceived stress. Participants completed the Rome IV scale on average 6 months (175 days, s.d.: 73.8) from their acute infection. Females (87.8% *vs.* 72.6%, *P* = 0.02), and those with a higher acute illness severity (6.7 *vs.* 5.8, *P* < 0.01) were significantly more likely to complete the Rome IV survey (Supplementary Table S1). Of the 49 participants who completed the Rome IV survey, 10 (20.4%) had persistent abdominal symptoms consistent with IBS with an onset less than 6 months, and 5 (10.2%) met the Rome IV diagnosis for PI-IBS. This results in an incidence of 3.0% for those with GI symptoms during acute infection. Overall, PI-IBS was reported on average 6.2 months (175 days, s.d.: 61.6) from acute infection. As shown in Table 4, of the 49 participants who completed the Rome IV survey, the most bothersome symptom reported was watery or mushy stools or having multiple bowel movements in a day (34.7%, 95% CI 22.5–49.3), followed by abdominal pain (18.4%, 95% CI 9.7–32.1) and bloating (16.3%, 95% CI 8.2–29.8) (Table 4). The majority of participants indicated their abdominal pain was worse with menstruation if they were female (51.4%, 95% CI 30.6–60.7), worse after eating (52.4%, 95% CI 37.1–67.2), and restricting to their usual activities (52.4%, 95% CI 37.1–67.2).

**Table 3.** Effect modification of gastrointestinal symptoms during acute infection and gastrointestinal symptoms ≥45 days post-acute infection by chronic condition status in adult Arizona CoVHORT participants who tested positive for COVID-19 (May 2020–October 2021)

	No acute GI symptoms		Acute GI Symptoms		
	*N* with/without ≥45 days GI symptoms	OR (95% CI)	*N* with/without ≥45 days GI symptoms	OR (95% CI)	ORs (95% CI) for acute GI symptoms within strata of chronic disease status
No chronic conditions	7/381	1.0	16/154	4.29 (2.45–7.53) *P* < 0.001	8.34 (2.91–23.94) *P* < 0.001
At least 1 chronic condition	16/550	1.18 (0.66–2.11) *P* = 0.57	29/296	4.27 (2.43–7.51) *P* < 0.001	3.18 (1.62–6.26) *P* *=* 0.001

Abbreviations: GI, gastrointestinal; OR, odds ratio; CI, confidence interval.

All ORs are adjusted for age, sex and perceived stress via the Perceived Stress Scale (PSS).

**Table 4.** New-onset irritable bowel syndrome (IBS) features of adult Arizona CoVHORT participants that completed the ROME IV survey *n* = 49, May 2020–October 2021

	*N* (%)	95% Confidence interval
Pain in Abdomen in last 3 months		
Never	8 (16.3)	8.2–29.8
Less than one day a month	4 (8.2)	3.0–20.2
One day a month	1 (2.0)	0.3–13.7
Two to three days a month	13 (26.5)	15.9–40.9
Once a week	4 (8.2)	3.0–20.2
Two to three days a week	9 (18.4)	9.7–32.1
Most days	7 (14.3)	6.8–27.5
Every day	2 (4.1)	1.0–15.4
Multiple times per day or all the time	1 (2.0)	0.3–13.7
Pain occurring close to bowel movement^a^	22 (52.4)	37.1–67.2
Change in stools with abdominal pain^a^	28 (66.7)	50.8–79.5
Change in frequency of bowel movements with abdominal pain^a^	24 (57.1)	41.5–71.5
Abdominal pain characteristics^a^		
Worse with menstruation^b^	19 (51.4)	30.6–60.7
Worse after eating	22 (52.4)	37.1–67.2
Restricting to usual activities	22 (52.4)	37.1–67.2
Continuous pain	7 (16.7)	8.0–31.6
Duration of 6 months or longer	18 (42.9)	28.5–58.5
Most bothersome symptom		
Abdominal pain	9 (18.4)	9.7–32.1
Watery or mushy stools, or having many bowel movements in a day	17 (34.7)	22.5–49.3
Hard stools or going several days without having a bowel movement	5 (10.2)	4.2–22.7
Bloating or belly looking unusually large	8 (16.3)	8.2–29.8
None of the above, but another symptom	10 (20.4)	11.2–34.3

a Frequency reporting outcome 30% of the time or more in the past 3 weeks.

b Question only asked for participants who identify as Female, *n* = 37.

## Discussion

In this study, we aimed to understand the prevalence of GI symptoms post-acute COVID-19 infection and the health experience of those who developed PI-IBS as determined by the Rome IV survey. We used data from a prospective, longitudinal cohort of COVID-19 cases who self-report health and wellbeing over time. We found that participants with acute GI symptoms had 4 times the odds of persistent GI symptoms ≥45 days from acute infection. This association was modified by pre-existing chronic conditions status; those without pre-existing conditions had 8 times the odds of persistent GI symptoms. The health experience of those experiencing GI symptoms post-acute infection was considerable, and included 42 participants (61.7%) who sought care from a provider for their symptoms. Of participants who completed the Rome IV survey, 5 (10.2%) met the diagnostic criteria for PI-IBS, and overall 30.6% had persistent symptoms consistent with an IBS diagnosis. Taken together, these results demonstrate that persisting GI symptoms are an important outcome following SARS-CoV-2 infection, and more research is needed to understand how pre-existing chronic conditions, and other factors, may modify this relationship in other samples.

The proportion of our cohort who experienced GI symptoms during acute infection was similar to other studies [12], and the type of symptoms described by participants was considerable. Our results show that GI symptoms persist after acute COVID-19 infection and can have substantial impacts to a person's quality of life. The proportion of participants who had persistent abdominal pain consistent with an IBS diagnosis is similar to the one other COVID-19 study which estimated the incidence of PI-IBS in 26.2% of patients [12]. However, our reference group was COVID-19 cases without GI symptoms, and Noviello *et al*. used COVID-19 negative controls. Our study design choice can be particularly useful during outbreak investigations, or when data on controls is not available. The benefit of our design is that the health experience of those seeking treatment for their infection may be more similar, and providers may be able to provide targeted recommendations in the future given acute infection symptomology. Findings in this report are particularly useful in understanding PASC and may help providers be more aware of this outcome for those patients who have GI symptoms during acute infection.

A limitation of our approach is that we did not ask those without GI symptoms during their acute infection to complete the Rome IV, however we did ask about persisting GI symptoms of every participant 45 days post-acute infection, and 23 (2.4%) of those without acute GI symptoms had GI symptoms post-acute COVID-19 infection. We were also unable to discern acute GI symptom severity and duration from acute infection severity overall in this study; this is an area of future work for the Arizona CoVHORT.

Our study is limited by the use of self-reported data which may impact the interpretation of the results. Self-reported data can be beneficial for understanding a participant's experience, and can be particularly useful with a new disease like COVID-19 which has a wide range of symptoms and outcomes. We have used structured questionnaires to reduce social desirability bias and recruited directly following testing and surveillance interviews to reduce recall bias. The majority of our participants have come from this recruitment strategy which makes the CoVHORT team more confident in our exposure classification strategy. However, there may still be issues with using just self-reported data, and we hope to expand our efforts for tracking hospitalisations, other health outcomes, and death among participants in the cohort in the future. Connecting with disparate datasets will allow us to understand the full burden of COVID-19 more completely, and provide a way for us to verify self-reported outcomes.

The Arizona CoVHORT has similar demographic characteristics to reported US COVID-19 cases with similar Hispanic, possibly more affluent, and more female participants, by about 10%. We have included a comparison of the demographics in the cohort to Arizona, the US, and US COVID-19 cases in the Supplemental Material to aid in understanding these differences further. Additionally, more females completed the Rome IV survey, and females are more likely to seek care for and be diagnosed with IBS [25, 26], so this may have influenced our estimates. While we did adjust for sex in our analyses, this is an area that requires further investigation. After adjusting for missingness in perceived stress we had a slightly smaller point estimate of 3.63 (95% CI 2.09–6.28). Perceived stress was lower overall after imputation, thus perceived stress during acute infection may impact persistent GI symptoms. After redefining the outcome variable to 180 days post-acute infection, the results remained similar to the adjusted primary analysis however we did observe a larger CI due to participant drop out at 6 months. Encouraging participant engagement through incentives may help to reduce participant drop out in longitudinal studies such as the Arizona CoVHORT.

In conclusion, our study describes persistent GI symptoms and the development of new-onset PI-IBS in persons with GI symptoms during COVID-19 acute infection. While less is known about why GI symptoms resolve in some COVID-19 patients during their acute infection, and not in others, this paper adds to our knowledge about PASC, with important impacts to providers' care recommendations. Our finding that 61.7% of participants sought medical care for their symptoms speaks to the long-term healthcare capacity that is needed to care for persons with PASC, on an already taxed healthcare system because of COVID-19. We continue to learn a great deal from this pandemic and while the toll of acute infection and its attendant morbidity and mortality is enormous, many patients are known to continue to suffer from a variety of long COVID symptoms. With the hopes of vaccines and treatments ending the acute disease tragedy, many of the survivors could be continually plagued by ongoing symptoms and we need to understand how to best manage them. Patients with persistent GI symptoms can experience considerable impacts to their quality of life, thus timely diagnosis of functional GI disorders and management of symptoms should be a priority for providers of COVID-19 patients experiencing these issues.

## Data Availability

Data will be made available upon reasonable request of the corresponding author.

## Supplementary material

For supplementary material accompanying this paper visit https://doi.org/10.1017/S0950268822001200. click here to view supplementary material
