# Supplementary material for: Persisting gastrointestinal symptoms and post-infectious irritable bowel syndrome following SARS-CoV-2 infection: results from the Arizona CoVHORT
Source: Epidemiol Infect. 2022 Jul 8;150:e136. doi: 10.1017/S0950268822001200 (PMC9343359; doi:10.1017/S0950268822001200)
Supplement: Supplementary file 1 [file hygsup.zip › S0950268822001200sup002.docx]

**Appendix Table 1.** Characteristics of adult Arizona CoVHORT participants who tested positive for COVID-19, May 2020-October 2021 stratified by Rome IV survey completion

| **Characteristic, n(%)** | **Did not Complete ROME IV (n=450)** | **Completed ROME IV (n=49)** | *p-value^a^* |
| --- | --- | --- | --- |
| Age (years), mean (SD) | 42.7 (15.8) | 42.3 (13.5) | 0.95 |
| Sex |  |  | **0.02** |
| Female | 331 (72.6) | 43 (87.8) |  |
| Male | 125 (27.4) | 6 (12.2) |  |
| Ethnicity |  |  | 0.65 |
| Hispanic | 109 (24.3) | 13 (26.5) |  |
| Non-Hispanic | 340 (75.7) | 36 (73.5) |  |
| Highest Education Level |  |  | 0.35 |
| 9-12^th^ grade, or GED | 17 (5.4) | 1 (3.0) |  |
| Some college (1-3 years) | 95 (29.9) | 12 (36.4) |  |
| College (4 years or more) | 101 (31.9) | 7 (21.2) |  |
| Post-graduate education | 104 (32.8) | 13 (39.4) |  |
| Annual Household Income |  |  | 0.11 |
| $0-$74,999 | 121 (41.6) | 18 (60.0) |  |
| ≥$75,000 | 170 (58.4) | 12 (40.0) |  |
| Perceived Stress Scale ^b^, mean (SD) | 18.5 (8.2) | 20.9 (7.4) | 0.07 |
| Acute Infection Severity ^c^, mean (SD) | 5.8 (2.3) | 6.7 (2.2) | **<0.01** |

Abbreviations: GI, gastrointestinal; SD, standard deviation; GED, General Education Degree.

^a^ Significant differences between the groups were assessed using chi-square, t-tests, or nonparametric alternatives (Fisher’s exact for sex and education) as appropriate, *p*<0.05 is considered significant and is bolded

^b^ The Perceived Stress Scale ranges from 0-40, scores 0-13 indicate low stress, 14-26 indicate moderate stress, and 27-40 indicate high stress

^c^ Acute infection severity ranges from 0-10, with 1 indicating very mild illness, and 10 indicating extremely serious illness

**Appendix Table 2.** Characteristics of adult Arizona CoVHORT participants compared to the population of Arizona, United States COVID-19 case-patients, and the United States population

| **Characteristic, n(%)** | **Arizona CoVHORT Sample in this Study %** | **Arizona Population**  **N=7.1 Million ^1^** | **US Population  N=329.5 Million ^1^** | **US COVID-19 Case-patients N=73.4 Million ^2^** |
| --- | --- | --- | --- | --- |
| Age (years), median (SD) | 43 (15.8) | 37.9 (0.2) | 38.2 (0.1) | - |
| Sex |  |  |  |  |
| Female | 66.3 | 50.3 | 50.8 | 53.2 |
| Male | 33.0 | 49.7 | 49.2 | 46.8 |
| Other | 0.7 | - | - | <0.1 |
| Ethnicity |  |  |  |  |
| Hispanic | 21.8 | 30.7 | 18.7 | 24.8 |
| Non-Hispanic | 78.2 | 70.3 | 81.3 | 75.2 |
| Bachelor’s Degree or Higher | 31.2 | 30.3 | 32.9 | - |
| Household Income (median, in $USD) ^3^ | - | $61,529 | $64,994 | - |
| ≥$75,000 | 58.8 | - | - | - |

Abbreviations: GED, General Education Degree.

^1^ Estimates from the US Census 2020
^2^ Estimates from US Centers for Disease Control and Prevention COVID-19 Case Surveillance, May 5, 2022. Age is collected as a categorical variable – the largest age group is 18-29 (21.3%) 50-64 (18.3%) 30-39 (16.9%) 40-49 (14.3%). Education and household income data are not publicly available.

^3^ Income is asked in a categorical variable in the Arizona CoVHORT so median household income is not possible to calculate
